# Current Findings on *Allium* Species with Melanogenesis Inhibitory Activity

**DOI:** 10.3390/plants14111635

**Published:** 2025-05-27

**Authors:** Mariangela Marrelli, Maria Pia Argentieri, Vincenzo Musolino, Carmine Lupia, Claudia-Crina Toma, Filomena Conforti, Vincenzo Mollace, Giancarlo Statti

**Affiliations:** 1 Department of Pharmacy, Health and Nutritional Sciences, University of Calabria, 87036 Cosenza, Italy; studiolupiacarmine@libero.it (C.L.); filomena.conforti@unical.it (F.C.); giancarlo.statti@unical.it (G.S.); 2 Laboratory of Pharmaceutical Biology, Department of Health Sciences, Institute of Research for Food Safety & Health IRC-FSH, University “Magna Græcia” of Catanzaro, 88100 Catanzaro, Italy; v.musolino@unicz.it; 3 Department of Pharmacy-Drug Sciences, University of Bari Aldo Moro, 70125 Bari, Italy; mariapia.argentieri@uniba.it; 4 Mediterranean Ethnobotanical Conservatory, Sersale, 88054 Catanzaro, Italy; 5 National Ethnobotanical Conservatory, Castelluccio Superiore, 85040 Potenza, Italy; 6 Department of Pharmacognosy, Faculty of Pharmacy, Western University “Vasile Goldiş” of Arad, L. Rebreanu Street, No. 87, 310048 Arad, Romania; claudiatoma2004@yahoo.com; 7 Laboratory of Pharmacology, Institute of Research for Food Safety and Health IRC-FSH, Department of Health Sciences, University Magna Græcia of Catanzaro, 88100 Catanzaro, Italy; mollace@unicz.it

**Keywords:** *Allium*, onion, garlic, melanogenesis, skin, dermatology, tyrosinase, phytochemicals

## Abstract

*Allium* genus (Amaryllidaceae) is widely distributed in the Northern hemisphere. Some species, including garlic and onion, have been used since ancient times as both food ingredients and medicinal plants. Many reviews deal with the chemical constituents, particularly the typical sulfur compounds, as well as with *Allium* pharmacological properties, such as antimicrobial, anti-inflammatory, antioxidant, and cytotoxic activities. The bibliographic search performed in this review is mainly focused on the potential role of *Allium* species in inhibiting melanogenesis, which has been mainly assessed through the evaluation of the inhibitory properties on tyrosinase, the key enzyme in melanin biosynthesis. Two well established models for identifying potential skin-whitening agents have been used to assess the anti-melanogenic effects of *Allium* species, the mushroom tyrosinase and the murine melanoma B16 cell line. Here, a literature search from Scopus, Web of Science, and PubMed databases has been performed using the keywords “*Allium*”, “tyrosinase”, “anti-melanogenic”, and “melanogenesis”, combined by means of Boolean operators. Based on selected inclusion criteria, 32 eligible papers have been selected. The aim of this systematic review is to offer an overview of the species for which the ability to affect melanogenesis has been demonstrated to date, highlighting a new and emerging perspective on the potential therapeutic use of *Allium* species. The biological properties of isolated pure compounds and the negative outcomes have been also considered.

## 1. Introduction

Human skin and hair color are mainly due to melanin pigmentation. Melanins are widely distributed pigments, present in animals, plants, bacteria, and fungi, and are complex polyphenol-like polymers with colors ranging from yellow to black [1,2]. Melanin biosynthesis is regulated by the tyrosinase enzyme [3,4].

A correct biological control of melanogenesis is due to a complex balance of different factors and mechanisms. The enzymes responsible for melanin biosynthesis became functional only after they have entered the melanosomes and they become activated in the endoplasmic reticulum. Such mechanism allows a normal pigmentation [5].

An altered melanin biosynthesis is related to several pigmentation disorders. While the mutation in the tyrosinase genes cause oculocutaneous albinism, characterized by a reduced melanin production, an excess in melanin production or its abnormal distribution may cause over-tanning, age spots (solar lentigo), and melasma [6].

Commercially available skin lightening agents, useful against these three dermatological disorders, are mainly tyrosinase inhibitors designed to reduce melanin content [6,7]. The goal of skin whitening is to lighten skin tone by reducing the concentration of the pigment melanin. However, many commonly utilized chemical skin-whitening agents contain toxic compounds such as mercury chloride or ammoniated mercury as the active ingredient. The presence of such hazardous chemicals has led to the search for safer whitening agents based on plant natural compounds, with the aim to find safe and affordable new tools [8].

Naturally occurring tyrosinase inhibitors belong to different classes of compounds, including phenolics, flavonoids, alkaloids, terpenes, steroids, fatty acids, and even coumarins [8,9]. The flavonoid quercetin, occurring in many fruits and vegetables, such as blueberries, cranberries, apples, and in plant waste material, such as onion peel or grape pomace, demonstrated potential beneficial properties in the treatment of several skin disorders, including the protection against UV radiation, the inhibition of melanogenesis, and the prevention of skin oxidation [10,11]. Consistently, many plant extracts have been reported to exert anti-tyrosinase activity till now [12,13,14,15,16,17,18].

*Allium* genus includes garlic and onion species, which have been consumed for many centuries as food ingredients [19,20] and as medicinal plants [21]. This genus belongs to the Amaryllidaceae family, and it includes more than a thousand accepted species, with a wide distribution, from the dry subtropics to the boreal zone [22], and whose main center of diversity is ranging from the Mediterranean Basin to Central Asia [23]. The two main species with a well-known culinary use are *Allium cepa* L. and *Allium sativum* L., the common onion and garlic, respectively. This genus is important from an economical point of view, as about 50 species are cultivated for different purposes. However, many wild species are also collected by the local populations and are used as culinary and medicinal plants [24,25], and even for ornamental purposes [21,26]. Several studies demonstrated that other *Allium* species are a rich source of secondary metabolites with a wide spectrum of biological activities. Numerous constituents have been identified, belonging to different classes of compounds, such as flavonoids, alkaloids, and sulfur compounds [27,28,29,30].

Moreover, the health benefits of both *Allium* extracts and isolated pure compounds have been investigated through in vitro, in vivo, and clinical studies for their potential use in the treatment of several pathological conditions [31,32], such as the metabolic syndrome [33,34,35,36], and for their interesting antioxidant [37], antimicrobial [38,39,40], cytotoxic [41,42,43,44], and anti-inflammatory properties [43]. The role of *Allium* edible species in cancer prevention has been explored as well [45,46].

The analysis of the literature allowed us to identify the studies specifically focusing on the inhibitory effects of *Allium* species on tyrosinase enzyme and thus melanogenesis. The aim of this review is to offer an overview of the species for which the ability to affect melanogenesis has been demonstrated to date, and to underline a new and emerging perspective on the potential therapeutic use of *Allium* species, which has not been deeply reviewed till now, to the best of our knowledge.

## 2. Methodology

The bibliographic investigation was conducted using the search engines Scopus, Web of Science, and PubMed, following the Preferred Reporting Items for Systematic Reviews and Meta-Analyses (PRISMA) guidelines [47]. The keywords “*Allium*”, ”tyrosinase”, “anti-melanogenic”, and “melanogenesis” were used and combined by means of the Boolean operators “and” and “or”.

This systematic review was performed through a machine-learning-based approach using the MySLR digital platform [48], available at https://myslr.unical.it (accessed on 21 January 2025). A methodological approach including different steps, namely manuscripts selection and analysis and the synthesis of the results, was adopted using this semi-automated tool.

**Figure 1:**
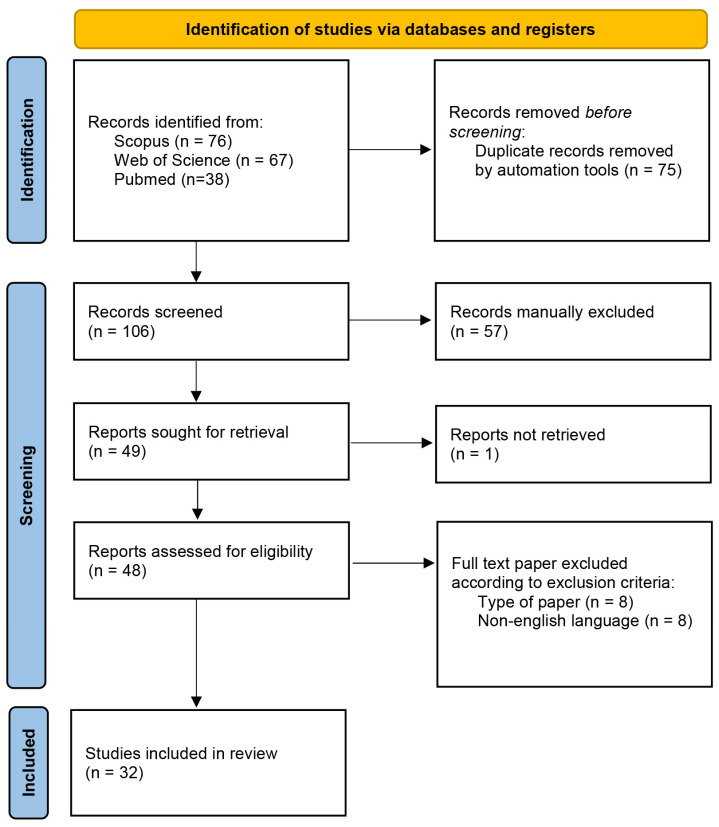
Selection process of the eligible reports based on the PRISMA 2020 flow diagram.

The selection process of the papers included in this review is illustrated in Figure 1.

Seventy-six studies were retrieved in Scopus, 67 in Web of Science, and 38 in PubMed database, accounting for 181 papers. Duplicates (75) were then removed and the titles and abstracts of the remaining 106 papers were checked in order to select those studies that fitted the following inclusion criteria: studies published in English, and articles whose title and/or abstract referred to the specific use of *Allium* species on tyrosinase and melanogenesis inhibition. The following exclusion criteria were adopted: papers not in English, previous reviews, book chapters, conference papers, and letters to editors were excluded.

After this manually performed screening, 57 bibliographic sources were excluded, and 1 full-paper was not retrieved. The remaining 48 full-text records were deeply inspected. Eight studies were excluded according to the type of paper, and another eight were excluded because they were not in English. Finally, 32 papers were included in this systematic review.

**Figure 2:**
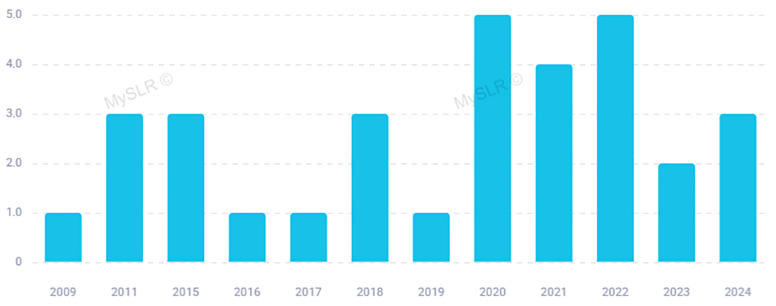
Distribution of selected papers by year of publication (Figure produced by MySLR platform).

The distribution over time of the selected 32 studies focused on the potential melanogenesis inhibitory activity of *Allium* species is illustrated in Figure 2. Starting from 2009 up to date, the peak number of publications occurred in 2020 and 2022, with 5 articles per year, overall, accounting for about 31% of the whole selection. No articles were published in 2010 and between 2012 and 2014 (Figure 2).

**Figure 3:**
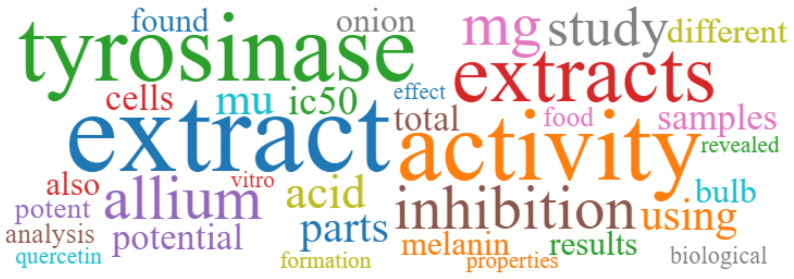
Word cloud highlighting the most important keywords (created by MySLR).

The most significant keywords are reported in Figure 3, in which they are visually represented through a “word cloud”.

## 3. Melanogenesis

### 3.1. Melanin Structure and Functions

In humans and other mammals, the intracellular pigment melanin is present in different organs including the skin, where its amount is responsible for skin color and it plays a pivotal role in the protection against DNA damage due to UV radiation [6,49].

Overall, in mammals, melanin is produced by melanocytes, eye pigment epithelium, and neurons, and it occurs in three forms: neuromelanin, eumelanin, and pheomelanin. The first two forms are brown-black, while the last one has golden-yellow-red color. The neuromelanin is present in the substantia nigra and the loci cerulea in the brain. Eumelanin is the main epidermal melanin, being responsible for the variable color of the skin, from fair to brown and dark, and the black, brown and blond color of the hair. Pheomelanin, which differs from eumelanin for its higher content of sulfur and cysteine, is instead present above all in red hair and in the skin of lips, nipples, and genitals. Brown, gray, green, and blue eye colors are due to the proportion of eumelanin, pheomelanin, and capillary blood [50].

### 3.2. Melanin Biosynthesis: The Tyrosinase Enzyme

Melanin biosynthesis, namely melanogenesis, occurs in response to ultraviolet B (UVB) irradiation in melanosomes present within melanocytes, specialized cells existing in the skin and hair, as well as in the retinal pigment epithelium cells in the eye. Melanin is then transported to the neighboring keratinocytes in the epidermis [7,51].

Two types of melanin are produced in melanocytes: eumelanin and pheomelanin. The melanin biosynthesis is regulated by the key enzyme tyrosinase (Tyr), only produced by melanocyte cells [7]. This enzyme shows two mechanisms of oxidation, namely monooxygenase and oxidase activities, and four oxidation states of the two copper atoms in the active site have been demonstrated, that is the oxy-, deoxy-, met-, and deact-tyrosinase [52]. Tyrosinase is also referred to as monophenol monooxygenases, as this key regulatory enzyme catalyzes the conversion of monophenols such as tyrosine into *o*-diphenols, and the subsequent oxidation to the corresponding *o*-quinone derivatives [6,7].

The first step of melanogenesis is the oxidation of L-tyrosine to dopaquinone by the key enzyme tyrosinase; dopaquinone is a precursor of eumelanin and pheomelanin. Dopaquinone undergoes cyclization to give indoline, leukodopachrome (cyclodopa). The redox exchange between leukodopachrome and dopaquinone produces dopachrome and L-3,4-dihydroxyphenylalanine (L-DOPA), which is also a substrate for the tyrosine enzyme and is again oxidized to dopaquinone [7].

**Figure 4:**
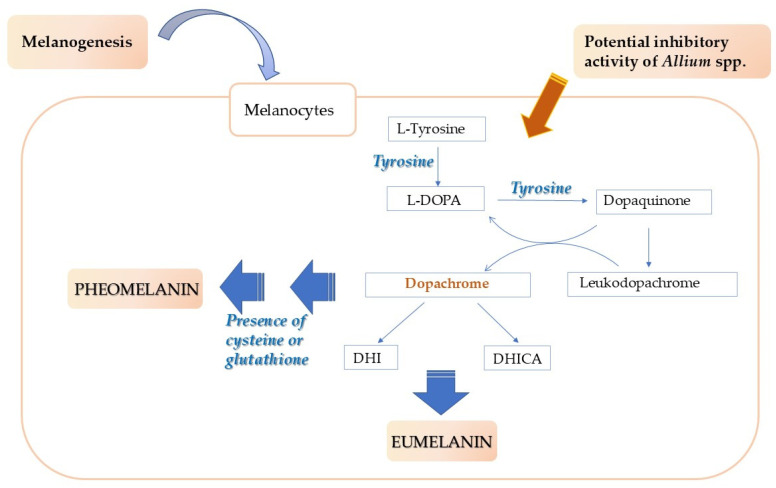
Scheme of the melanin biosynthesis and investigated role of *Allium* species.

Both in plants and animals, tyrosinase can catalyze the hydroxylation of tyrosine to L-3,4-dihydroxyphenylalanine (L-dopa), which is then converted into L-dopaquinone. However, differently from plants, in humans, two more enzymes called tyrosinase-related proteins, Tyrp1 and Tyrp2, are involved, allowing a refined control of the biosynthesis (Figure 4) [53].

The synthesized dopachrome is gradually converted into dihydroxyindole-2-carboxylicacid (DHICA) and dihydroxyindole (DHI). The formation of DHICA is catalyzed by Tyrp2. Finally, the two dihydroxyindoles DHICA and DHI are oxidized to eumelanin. It is supposed that Tyrp-1 may catalyze the oxidation of DHICA to eumelanin. Moreover, in the presence of cysteine or glutathione, dopaquinone is also converted into 5-S-cysteinyldopa or glutathionyldopa, which undergoes subsequent oxidation to finally give pheomelanin [7].

The formation of dopaquinone is the rate-limiting step in the biosynthesis of melanin, as the other steps can proceed spontaneously. So, even if three enzymes, tyrosine, Tyrp-1, and Tyrp-2, are involved in melanogenesis, tyrosinase is the only key enzyme of the process [7,54].

The tyrosinase enzyme also catalyzes the neuromelanin synthesis, in which the oxidation of dopamine gives dopaquinones [7].

## 4. Tyrosinase Inhibitors from Plants

Given the key role of the tyrosinase enzyme in melanin biosynthesis, the tyrosinase inhibitors are the most useful tool to inhibit melanogenesis. This mechanism of action also allows avoiding side effects, as the tyrosinase enzyme is only produced by melanocytes. Many tyrosinase inhibitors, such as kojic acid, azelaic acid, and ellagic acid, have been used as skin-whitening agents [7,55,56].

The inhibitory properties of plant extracts and pure compounds are usually assessed in vitro using mushroom tyrosinase enzyme from *Agaris bisporus*, considered a well-established model for identifying potential skin-whitening agents, and using active compounds such as kojic acid or hydroquinone as positive controls [57]. The effectiveness in inhibiting melanogenesis is usually also verified on the murine melanoma B16 cell line, in which the same melanogenesis mechanism of normal human melanocytes has been demonstrated. Among secondary metabolites from plant source, different classes of compounds showed effectiveness, such as flavanones and chalcones and coumarin derivatives [7].

With the aim of finding tyrosinase inhibitors with strong activity and low side effects, there is a growing interest in plants and their metabolites. Different flavonoids, such as quercetin and kaempferol, demonstrated a strong tyrosinase inhibitory property, as well as some gallic acid derivatives. Moreover, a wide number of aldehydes and other phytochemicals have been demonstrated to inhibit the tyrosinase enzyme, such as *trans*-cinnamaldehyde, 3,4-dihydroxycinnamic acid, and 4-hydroxy-3-methoxycinnamic acid [57].

## 5. *Allium* spp. Extracts with Tyrosinase Inhibitory Potential

**Figure 5:**
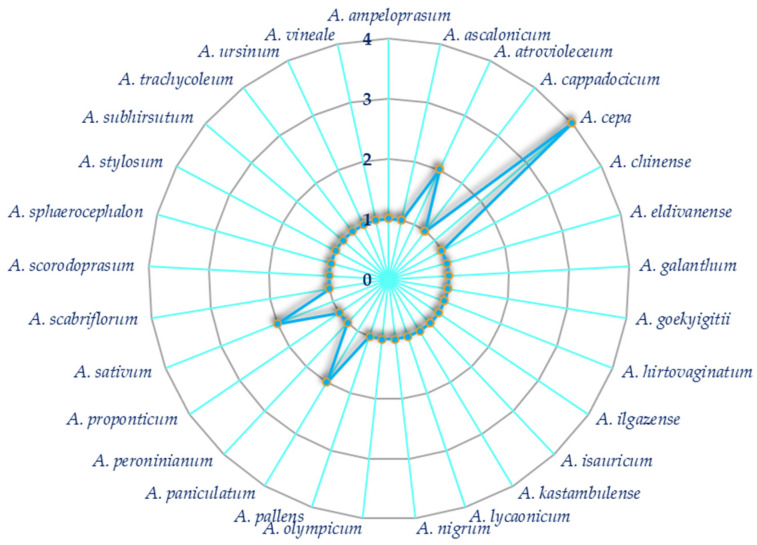
Kiviat diagram visualizing the number of studies focusing on each investigated *Allium* species.

Several *Allium* species have been investigated to date as regards their ability to affect melanogenesis by inhibiting the tyrosinase enzyme. Overall, as illustrated in Figure 5, onion (*A. cepa* L.) is the most investigated species, followed by garlic (*A. sativum* L.), *A. atrovioleceum* Boiss., and *A. paniculatum* L.

Emir and coworkers investigated the enzyme inhibitory potential of *A. ampeloprasum* L. methanolic extracts. Samples from the flower, leaf, and bulb of the plant showed inhibitory properties on mushroom tyrosinase enzyme, with IC_50_ values equal to 207.85, 313.40, and 348.10 μg/mL, respectively [58].

Phetmanee and coworkers assessed the anti-melanogenic properties of shallots (*A. ascalonicum* L.) from 14 different cultivation sites in Thailand, extracted with both water and ethanol. At the concentration of 1 mg/mL, the samples showed about 10–15% tyrosinase inhibition. An optimized shallot sample from fresh shallots collected from Phayao, extracted with 20% ethanol at 40 °C, was demonstrated to decrease melanin biosynthesis in B16F10 cells in a concentration-dependent manner, without affecting cell viability [59] (Table 1).

Emir and colleagues also assessed the tyrosinase inhibitory potential of different methanolic extracts from the bulb, stem and flower of *A. atrovioleceum* Boiss. The plant was collected in two different localities in Turkey and the different obtained extracts were tested on mushroom tyrosinase. A good biological potential was observed, with IC_50_ values equal to 62.53, 67.40, and 78.83 μg/mL for the flower, bulb, and stem extracts of the most effective sample, collected in Kemalpaşa, İzmir, Turkey [60].

The same *Allium* species, *A. atrovioleceum* Boiss., was also investigated by Rocchetti and colleagues. Aerial parts and bulb were extracted through both maceration with methanol and hot water infusion. All the samples showed tyrosinase inhibitory activity, with the methanolic extracts (43.86 and 43.44 mg KAE/g for the aerial parts and bulb, respectively) showing better activity compared to the water extracts (28.78 for the aerial parts and 20.36 mg KAE/g for the bulb) [61].

The same authors also performed this kind of experiments on another *Allium* species, *A. cappadocicum* Boiss. and Balansa. The methanolic macerate and the water infusion of both aerial parts and bulb were effective in inhibiting tyrosinase, with MeOH extracts (48.63 and 49.51 mg KAE/g for the aerial parts and bulb, respectively) showing higher activity compared to the water extracts (28.50 for the aerial parts and 29.22 mg KAE/g for the bulb) [61].

As illustrated in Figure 5, *A. cepa* L. is the most investigated *Allium* species with respect to the anti-tyrosinase activity. Arung and coworkers investigated the biological potential of the methanolic extract of the dried skin and flesh of red onion *A. cepa.* The studied plant was bought from a traditional market in Jakarta, Indonesia, and both powdered dried skin and flesh part were extracted with methanol. The inhibitory effects on melanogenesis were evaluated in vitro on B16 mouse melanoma cells. The dried skin extract induced a concentration-dependent inhibition of the melanin production in B16 melanoma cells, which was evident at concentrations of 50 and 100 μg/mL, while no effects were detected for the flesh extract even at higher concentrations [62].

Jeong and coworkers investigated a different kind of extract from *A. cepa*, obtained from onions first fermented with *Saccharomyces cerevisiae* and then extracted with methanol. The sample was tested on B16F10 murine melanoma cells incubated with a concentration of 100 μg/mL for 24 h. *A. cepa* extract decreased the protein level of cellular tyrosinase to 65.82% [63].

The anti-tyrosinase potential of *A. cepa* was also investigated by Nile and colleagues. The authors considered the red onion solid waste: the outer dry skins and basal and apical trimmings of red onions bulb were pulverized and extracted with 80% ethanol. The obtained extract was also partitioned using different solvents. All the fractions were evaluated and proved effectiveness, with IC_50_ values ranging from 38.9 to 65.9 μg/mL. The 80% aqueous methanol extract was the most effective sample (IC_50_ = 38.9 μg/mL), followed by 80% aqueous ethanol and diethyl ether fractions (IC_50_ = 40.8 and 48.3 μg/mL) [64].

Tyrosinase is responsible not only for the production of melanin in animals, but also for browning in plants. The role of some *Allium* species has been also taken into account in the management of browning in fruits and vegetables. The browning reactions which occur during processing and storage are a major cause of post-harvest loss of vegetables and fruits. Enzymatic browning is due to some enzymes such as peroxidase, polyphenol oxidase, and phenylalanine ammonia-lyase. Among these enzymes, the one mainly involved in enzymatic browning reaction is polyphenol oxidase, commonly referred to as monophenol oxidase (MPO), diphenol oxidase (DPO), phenolase, and tyrosinase [80].

Tinello and colleagues evaluated the potential effectiveness of onion (*A. cepa* L.) juice and distillates in inhibiting enzymatic browning, assessing their inhibitory properties on both a commercial mushroom tyrosinase and some plant polyphenol oxidase (PPOs). The authors tested extracts and distillates from the inner layers of white, yellow, and red cultivars and Borettane onions. Among tested samples, white onion distillate, red onion juice, and yellow onion juice showed the best anti-tyrosinase activity, with inhibition values equal to 41%, 37%, and 37%, respectively [65].

Yu and colleagues isolated an effective saponin fraction from the 60% ethanolic bulb extracts of *A. chinense* G.Don. The fraction was demonstrated to inhibit the tyrosinase activity in B16 cells and to decrease the melanin biosynthesis [66].

Recently, Ozel and colleagues investigated the anti-tyrosinase potential of the methanolic extracts from the aerial parts of four different *Allium* species: *A. eldivanense* Özhatay, *A. ilgazense* Özhatay, *A. olympicum* Boiss., and *A. peroninianum* Azn. Samples were tested in vitro on mushroom tyrosinase, and a promising inhibitory effect was observed for *A. eldivanense* sample, for which an IC_50_ value equal to 11.87 µg/mL was obtained. The other *Allium* extracts were also proven to be effective, with IC_50_ values equal to 64 µg/mL (*A. ilgazense*), 128 µg/mL (*A. peroninianum*), and 321 µg/mL (*A. olympicum*) [67].

Kadyrbayeva and colleagues tested the tyrosinase inhibitory properties of different extracts from *A. galanthum* Kar. and Kir. The bulb and the chives of the plant were extracted with different solvents and techniques. Absolute ethanol, 70% ethanol, 50% ethanol, and water extracts were obtained by ultra-sound-assisted maceration. Diethyl ether extracts were also prepared. The efficacy of this species was tested on both mushroom and murine tyrosinase using the B16F10 cell line. The 50% and 75% ethanol extracts from the chives significantly inhibited murine tyrosinase in B16F10 cells. On the contrary, the aqueous and ethanolic extracts from the bulb were not effective on tyrosinase activity, except for the 96% ethanol sample (inducing 25% inhibition of mushroom tyrosinase at 100 μg/mL). The most active murine tyrosinase inhibitor was the diethyl ether extract from the bulb (which induced 82.65% inhibition of mushroom tyrosinase and decreased the activity of murine tyrosinase by 54% at 100 μg/mL) [68].

The activity of the methanolic and water extracts from the bulb and aerial parts of *A. goekyigitii* Ekim, H.Duman, and Güner was verified as well. The methanolic macerate and the water infusion of both aerial parts and bulb were effective in inhibiting tyrosinase, with MeOH extracts (51.17 and 49.70 mg KAE/g for the aerial parts and bulb, respectively) showing higher activity than the water extracts (25.72 for the aerial parts and 16.72 mg KAE/g for the bulb) [61].

Rocchetti and colleagues also verified the anti-tyrosinase potential of *A. hirtovaginatum* Kunth. Both aerial parts and bulb of the plant were extracted with maceration using methanol and through infusion. All the four obtained samples were tested in vitro with positive results. The macerates showed the most interesting results (49.53 and 46.50 mg KAE/g for the aerial parts and bulb, respectively), followed by the water extracts (26.26 for the aerial parts and 19.93 mg KAE/g for the bulb) [61].

The same experiments were also performed on the extracts obtained with the same techniques from aerial parts and bulb of *A. isauricum* Hub.-Mor. and Wendelbo. Samples showed tyrosinase inhibitory activity, with MeOH extracts (52.84 and 51.07 mg KAE/g for the aerial parts and bulb, respectively) showing higher activity compared to the extracts obtained through infusion (25.41 for the aerial parts and 17.25 mg KAE/g for the bulb).

Kisa and coworkers tested the inhibitory effects on mushroom tyrosinase of an extract from the species *A. kastambulense* Bosse. The aerial parts were extracted with a solution of methanol/chloroform (4:1), and the obtained sample was able to inhibit the enzyme with an IC_50_ value equal to 59.17 µg/mL [69].

Different extracts from *A. lycaonicum* Siehe ex Hayek were also evaluated for their anti-tyrosinase effects. Both bulbs and aerial parts were extracted with two extraction techniques, namely maceration and soxhlet apparatus, and using three different solvents, *n*-hexane, methanol, and water. All the samples showed in vitro inhibitory properties on tyrosinase, with the methanolic extracts being the most effective ones (values equal to 139.40 and 132.39 mg KAE/g for the aerial parts and bulb macerates, and values of 138.95 and 139.95 mg KAE/g for the extracts obtained with Soxhlet) [70].

The methanolic extracts from the bulb and aerial part of the species *A. nigrum* L. demonstrated good inhibitory properties on mushroom tyrosinase enzyme, with IC_50_ = 22.31 and 51.66 μg/mL for the aerial parts and bulb extracts, respectively [71].

Emir and coworkers assessed the inhibitory potential of *A. pallens* L., collected in two different localities in Turkey. The bulb, stem, and flower part of the plant were extracted with methanol. The most active samples demonstrated good inhibitory activity, with IC_50_ values equal to 54.58, 96.65, and 138.43 μg/mL for the stem, bulb, and flower extract, respectively [72].

Rocchetti and coworkers reported the biological potential of *A. paniculatum* L. The methanolic extracts showed an interesting tyrosinase inhibitory activity, with values equal to 52.87 and 53.17 mg KAE/g for the aerial parts and bulb, respectively, while a lower activity was observed for the samples obtained through infusion (6.35 and 3.02 mg KAE/g) [61].

Two subspecies of *A. paniculatum* L. collected in Turkey demonstrated tyrosinase inhibitory potential: *A. paniculatum* L. subsp. *paniculatum* L. and *A. paniculatum* L. subsp. *villosulum* (Hal.) Stearn. Three parts of the plants, namely bulbs, stems, and flowers, were extracted with methanol and chemically and biologically characterized. All the samples from the subsp. *villosulum* were effective, with IC_50_ = 49.16 μg/mL for the stem, 85.93 μg/mL for the bulb and 114.25 μg/mL for the flower extract. Except for the stem sample, which was not effective, also the extracts from *A. paniculatum* L. subsp. *paniculatum* L. showed tyrosinase inhibitory activity (IC_50_ values of 73.82 and 139.41 μg/mL for the flower and bulb samples, respectively) [73].

Interestingly, Emir and colleagues evaluated the effects of tyrosinase of the essential oil from the aerial parts of the species *A. proponticum* Stearn Et N. Özhatay subsp. *proponticum* Stearn Et N. Özhatay, an endemic species in Turkey. The essential oil was shown to inhibit mushroom tyrosinase (IC_50_ = 38.22 μg/mL) [81].

Somman and colleagues evaluated the inhibitory potential on tyrosinase enzyme of the species *A. sativum* L., considering both 80% methanol extract from fresh garlic and a processed garlic. This last preparation was preserved as a syrup using water with sugar, salt, and vinegar. Both fresh and processed garlic showed inhibitory potential (from 90.88% inhibition to higher values per 100 g) [74].

The biological activity of the species *A. sativum* was also studied by Samdavid Thanapaul and coworkers, who reported the evaluation of a multi-herbal formulation also containing *Coriandrum sativum* L., *Curcuma longa* L., *Mentha piperita* L., *Piper nigrum* L., *Syzygium aromaticum* (L.) Merr. and L.M. Perry, *Syzygium cumini* (L.) Skeels, and *Trigonella foenum-graecum* L., Murraya koenigii (L.) Spreng. The authors reported that the preparation inhibited the enzyme with an IC_50_ value of 252.87 μg/mL [75].

Rocchetti and coworkers tested different *Allium* species from Turkey for their enzyme inhibitory properties, including *A. scabriflorum* Boiss. Both aerial parts and bulb were extracted with two extraction techniques: maceration with methanol and hot water infusion. All the samples showed tyrosinase inhibitory activity, with MeOH extracts showing higher activity (44.89 and 43.73 mg KAE/g for the aerial parts and bulb, respectively) compared to the water extracts (29.48 for the aerial parts and 23.42 mg KAE/g for the bulb) [61].

Mollica and colleagues reported the tyrosinase inhibitory properties of methanolic extracts from *A. scorodoprasum* L. subsp. *rotundum* (L.) Stearn. The flower sample was the most effective one (55.21 mg KAE/g extract). Inhibitory properties were also detected for the stem and bulb extracts, with values equal to 49.23 and 38.27 KAE/g extract, respectively [76].

Emir and colleagues also evaluated the tyrosinase inhibitory potential of two subspecies of *Allium sphaerocephalon* L, namely *A. sphaerocephalon* L. subsp. *sphaerocephalon* L. and *A. sphaerocephalon* L. subsp. *trachypus* (Boiss. Et Spruner) K. Richter. The bulb, stem, and flower part of the two plants were extracted with methanol and the resulting extracts were investigated in vitro for their inhibitory potential on mushroom tyrosinase. The best activity was obtained for the bulb extract from the subsp. *sphaerocephalon* L., with an IC_50_ value equal to 65.94 μg/mL. Except for the flower extract from the subspecies *trachypus*, all the samples were effective. IC_50_ values of 179.42 and 204.71 μg/mL were obtained for the flower and stem extracts from *A. sphaerocephalon* L. subsp. *sphaerocephalon* L., and values equal to 262.50 and 315.88 μg/mL were reported for the stem and bulb extracts of the second subspecies [77].

Another study assessed the biological properties of the Turkey endemic species *A. stylosum* O. Schwarz. Also in this case, various parts of the plant and different sites of collection were considered. The bulbs, leaves, and flowers were extracted with methanol and the resulting samples were tested for their anti-tyrosinase activity on the mushroom enzyme. For the most active samples, collected in Bayramli, Izmir, IC_50_ values equal to 49.87, 75.97, and 170.35 μg/mL were obtained for the leaf, flower, and bulb extracts, respectively [78].

The inhibitory potential of the species *A. subhirsutum* L. was investigated as well. Both the bulbs and aerial parts were extracted with methanol and tested in vitro on the mushroom tyrosinase enzyme. A good biological activity was observed for the bulb and aerial part samples, with IC_50_ = 49.21 and 63.77 μg/mL, respectively [71].

Furthermore, the aerial parts and the bulb from *A. trachycoleum* Wendelbo were investigated. Samples were extracted with methanol through maceration, and a second extraction technique was also utilized, namely the hot water infusion of both plant parts. All the samples showed tyrosinase inhibitory activity, with the methanolic extracts (51.23 and 48.70 mg KAE/g for the aerial parts and bulb, respectively) showing higher activity compared to the water infusion extracts (27.33 for the aerial parts and 23.86 mg KAE/g for the bulb) [61].

The species *A. ursinum* L. was also effective. The leaves were extracted with three different solvents, namely water, 70% ethanol, and absolute ethanol. The best activity was observed for the 70% ethanol sample, with an IC_50_ value = 0.392 mg/mL [79].

Rocchetti and colleagues also assessed the anti-tyrosinase potential of the species *A. vineale* L. The methanolic extracts of the aerial parts and bulb showed inhibitory properties on mushroom tyrosinase, with values of 49.67 and 48.41 mg KAE/g, respectively. Values of 12.14 and 5.30 mg KAE/g were reported for the infusion extracts [61].

## 6. Isolated Compounds from *Allium* Species with Inhibitory Effects on Melanogenesis

**Figure 6:**
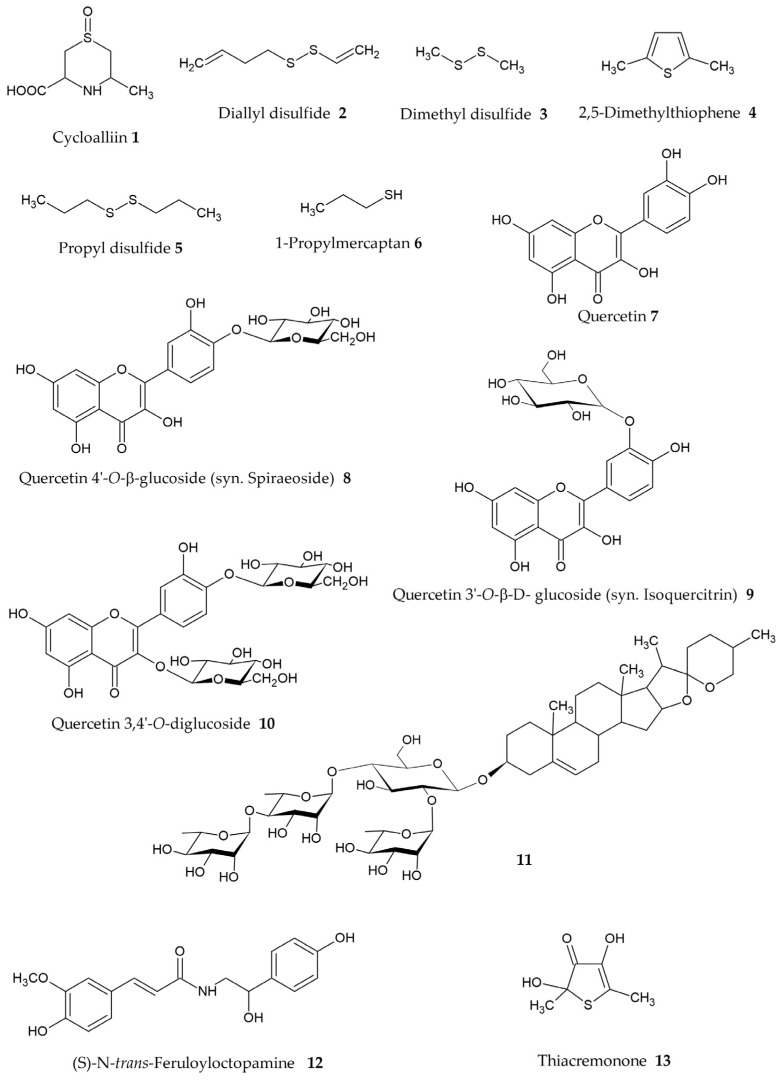
*Allium* chemical constituents showing melanogenesis inhibitory properties (chemical structures were drawn using ACD/ChemSketch (Freeware) 2024.2.0).

Beside the studies focusing on the activity of whole *Allium* crude extracts, some works dealt with the evaluation of pure isolated chemical constituents. Given the abundance of sulfur compounds in plants belonging to the *Allium* genus, this class of compounds is also the most investigated one as regards the inhibition of melanogenesis, with six molecules having been studied (Table 2, Figure 6).

Bito and colleagues evaluated the inhibitory property on melanin biosynthesis of cycloalliin (**1**, Figure 6), a sulfur-containing amino acid typically found in garlic and onion [89,90]. In this study, cycloalliin was obtained by synthesis, and it was tested in vitro for its inhibitory properties on both mushroom tyrosinase and B16 cell line. Just a weak concentration-dependent inhibition of the enzyme was observed, with this sulfur compound showing a mixed inhibitory effect on the enzyme activity: the K_i_ values were calculated to be 56.0 and 13.6 mM for the monophenolase and diphenolase activities of tyrosinase, respectively. The authors also assessed the biological properties of cycloalliin on B16 cell line. This compound significantly reduced the α-melanocyte-stimulating hormone (α-MSH)-induced melanin levels at a final concentration of 3.4 μM. Both protein and mRNA levels of tyrosinase were affected as well [82].

Chu and coworkers investigated the biological properties of other five organo-sulfur compounds commonly found in *Allium* species [91,92,93]. The inhibitory properties were determined on tyrosinase enzyme using L-DOPA as substrate and on B16 cell line cultures. Dimethyl disulfide (**3**) was effective in inhibiting mushroom tyrosinase, with an IC_50_ value equal to 6.5 mM, and in inhibiting tyrosinase activity and melanin formation in B16 cells (40.57% and 20.77%, respectively, at a concentration of 500 μM) [83]. A minor potential was instead observed for diallyl disulfide (**2**), 2,5-dimethylthiophene (**4**) and propyl disulfide (**5**), with low inhibition percentages on tyrosinase enzyme at the concentration of 10 mM (ranging from 3.9% to 8.4%). A moderate activity was observed on B16 cells: at the concentration of 500 μM, they were able to inhibit melanin formation and tyrosinase activity with percentages equal to 15.61% and 24.35% for diallyl disulfide (**2**), 14.62% and 24.79% for propyl disulfide (**5**) and 15.61% and 35.77% for 2,5-dimethylthiophene (**4**) [83].

The compound 1-propylmercaptan (**6**) was able to inhibit the enzyme with an IC_50_ value equal to 0.5 mM. It also inhibited melanogenesis in B16 cells: at the concentration of 500 μM, it inhibited melanin formation and tyrosinase activity with percentages equal to 24.15% and 46.89%, respectively. The inhibitory kinetics on tyrosinase were analyzed by a Lineweaver-Burk plot, and authors reported that compound **6** worked as mixed-type inhibitor of tyrosinase activity. The kinetic study suggested that 1-propylmercaptan was able to reduce the affinity of the substrate for the enzyme, but it did not bind to the active site [83].

Beside the evaluation of the whole *A. cepa* extract, Arung and coworkers also tested the inhibitory properties on melanogenesis of some pure isolated compounds [62]. Quercetin (**7**) and quercetin 4′-*O*-β-glucoside (**8**) were effective in inhibiting the melanin formation in B16 melanoma cells, with IC_50_ values equal to 26.5 and 131 μM, respectively [62].

The compound quercetin 4′-*O*-β-glucoside (**8**) is also a synonym of quercetin 4′-*O*-β-D-glucopyranoside and spiraeoside. Arung and coworkers also investigated the anti-melanogenic properties of this flavonoid on the mushroom tyrosinase enzyme, and demonstrated that it inhibited the enzyme activity with IC_50_ values = 4.3 and 52.7 μM using L-tyrosine or L-DOPA as substrates, respectively [84].

From the same plant source, i.e., the methanolic extract of *A. cepa* L. dried skin, the compound quercetin-3′-*O*-β-D-glucoside (**9**) (a synonym of isoquercitrin) was also successfully tested. This molecule was evaluated for its inhibitory properties on melanin formation in B16 melanoma cells, and an IC_50_ value = 38.8 μM was observed. Moreover, it was demonstrated to inhibit the mushroom tyrosinase in vitro, with IC_50_ values equal to 6.5 μM and 48.5 μM using L-tyrosine and L-dihydroxyphenylalanine (L-DOPA) as substrates, respectively [85].

Nile and coworkers reported a tyrosinase inhibitory activity also for the flavonoid quercetin-3, 4′-*O*-diglucoside (**10**), which was identified and isolated from the outer dry skins and basal and apical trimmings of red onions bulb *A. cepa* L. The molecule effectively inhibited the enzyme with an IC_50_ value equal to 12.6 μM [64].

Kim and coworkers described the isolation of a spirostane-type steroidal saponin (**11**) from the root extracts of *A. hookeri* Thwaites. The plant was extracted with 80% methanol and the compound was identified through NMR spectroscopic methods. This saponin showed inhibitory activity on mushroom tyrosinase with an IC_50_ value equal to 248.7 μM [86].

Wu and colleagues tested the inhibitory effects of a further phenolic constituent isolated from the garlic skin, (S)-N-*trans*-feruloyloctopamine (**12**). This molecule decreased the melanin content in α-MSH-stimulated B16F10 cells in a dose-dependent manner. Real-time PCR and Western blot analyses demonstrated that it down-regulates mRNA and protein expression levels of tyrosinase, leading to a lower melanin content [87].

Finally, the alpha-hydroxy ketone thiacremonone (2,4-dihydroxy-2,5-dimethyl-thiophene-3-one, **13**) was identified as a tyrosinase inhibitor. This compound was isolated from the heated garlic juice treated at 130 °C for 2 h by Woo and colleagues. The molecule inhibited the enzyme with an IC_50_ value equal to 101.931 μg/mL [88].

## 7. Negative Outcomes

As regards *A.cepa* L. extracts, some negative results were reported by Arung and coworkers. The authors, while demonstrating a good biological activity for the methanolic extract of red onion dried skin, reported that no effects were observed on melanin formation in B16 cells for the flesh extract from the same species [62]. The authors hypothesized that the differences in the melanogenesis inhibitory activity were related to the difference in the phytochemical content of onion skin and flesh, particularly the different abundance of quercetin and its derivative. HPLC analyses performed on the dried skin extract revealed contents of quercetin, isoquercitrin, quercetin 4′-O-glucoside, and quercetin 3,4′-O-diglucoside equal to 13.8%, 10.3%, 6.4%, and 8.3%, respectively. In the flesh extract, the amount of these compounds was significantly lower, with percentages equal to 0.01%, 0.59%, 0.35%, and 0.50%, respectively [62].

Any inhibitory effect on mushroom tyrosinase enzyme was detected for *Allium ascalonicum* L. peel extract [94], as well as for *A. flavum* L. hydroalcoholic extracts [95].

A negative outcome was also reported for the aqueous extract from the bulb of *A. turkestanicum* Regel, a Kazakh onion species. The sample, tested on B16F10 cells, did not show inhibitory properties and even increased the activity of murine tyrosinase [68] (Table 3).

## 8. Concluding Remarks and Future Perspectives

It has been recently highlighted that a consistent percentage of the world population, about 15%, invest in skin-whitening agents, with Asia being the main consumer [96,97]. Depigmenting agents may be divided based on the interference in melanin synthesis, transport, and removal by skin turnover [98].

Given the pivotal role of tyrosinase enzyme in melanogenesis, the inhibition of tyrosinase enzyme is the main extensively studied approach to prevent dark spot formation. However, depigmentation agents may also use other mechanisms of action, such as the epidermal turnover accelerant (glycolic acid, salicylic acid, liquiritin), the inhibitors of melanosome transfer (e.g., linoleic acid, which is able to block melanogenesis by preventing the transfer of melanosomes from melanocytes to keratinocytes), or free radical trapping agents (e.g., topical steroids and glycyrrhetinic acid) [55]. For example, the amide alkaloid piperlongumine, isolated from *Piper longum* L., is able to affect melanogenesis without inhibiting tyrosinase enzyme, but because of its ability to inhibit the α-MSH-induced melanogenesis. The endogenous tridecapeptide neurohormone α-MSH modulates inflammatory cutaneous and immune responses in normal human keratinocytes, melanocytes, and dermal fibroblasts, and it is the most important hormone which stimulates melanocytes in melanogenesis [99]. However, the inhibition of tyrosinase activity is still one of the main targets for reducing the melanin production. As a consequence, the tyrosinase inhibitors are the most used commercially available skin-lightening agents. Nevertheless, some of these active principles, such as hydroquinone or even the often utilized kojic acid, may cause a number of harmful side effects, which have led to some restrictions in their application [97]. Hydroquinone, the world’s most common whitening raw material, is a hydroxyphenol naturally present in plants such as cranberries and blueberries [100] and it has been banned in Europe in 2001 for its association with cancer [97]. Some European commercial preparations more recently introduced contain a combination of the derivative hydroquinone monomethyl ether (MEHQ, also known as 4-hydroxyanisole or mequinol), with retinoic acid. This combination shows a synergist effect without inducing side effects [55]. However, because of its teratogenicity, retinoic acid cannot currently be used in cosmetic products, but just as a prescription medicine in clinical dermatology [101].

Kojic acid (5-hydroxy-2-hydroxymethyl-4H-pyran-4-one) is a natural compound commonly used as a skin depigmenting agent. This organic acid is derived from the fermentation of fungi of diverse genus such as *Aspergillus* and *Penicillium* [102]. It has been established that the density of kojic acid in cosmetics must be lower than 1%, as the long-term application of this compound may cause skin sensitization [97].

Given the recent trend towards “green chemistry” and the choice for “green cosmetics”, natural raw plant materials are currently receiving great attention, as well as other natural and environmentally friendly bioactive compounds from marine algae and some common microorganisms [15,97,103,104].

Other natural products from plants already used as skin-whitening agents are ascorbic acid (Vitamin C) and its derivatives, arbutin and aloesin. Arbutin is a hydroquinone glycoside commonly found in Ericaceae, such as bearberry and strawberry tree, Apiaceae, Rosaceae, and Lamiaceae [55], while aloesin is a chromone derivative isolated from *Aloe vera*, known for its wound and burn healing properties, anti-inflammatory, and immunomodulatory effects [105]. Some plant extracts are also currently used in some commercial lighting products, such as *Morus alba* L. leaf extract and root extract, and *Glycyrrhiza glabra* L. root extract [55,99].

Among the different classes of natural compounds, the role of flavonoids in melanogenesis has been particularly investigated. It has been demonstrated that some of these phytoconstituents, such as cyanidin, hesperetin, and apigenin, are able to stimulate melanogenesis, while other flavonoids, including hesperidin, luteolin, and kaempferol exhibit anti-melanogenic effects [99]. Glabridin, a prenylated isoflavan from the roots of *Glycyrrhiza glabra* L. [106], acts as a tyrosinase inhibitor [99].

Skin-whitening agents are used both by dermatologists for the treatment of hyperpigmented lesions, and also by the public for cosmetic use [100]. Beyond the pharmacological aspects, the food industry is also interested in tyrosinase inhibitors, as the activity of the enzyme is responsible for the browning of fruit and vegetables. Cysteine or ascorbic acid, for example, is used to prevent the enzymatic browning by binding the dopaquinone intermediates [98].

The skin-whitening potency of a plant extract or a pure compound may be verified by several methodologies, ranging from in vitro assays to in vivo and clinical studies [107].

This review was designed with the aim of offering an overview of the species belonging to the *Allium* genus for which some anti-melanogenic properties have been highlighted. Overall, 32 papers were included. The in vitro activity of a total of 29 *Allium* species has been investigated till now. Moreover, the efficacy, at different extent, of 13 pure chemical constituents present in these species has been described as well. Onion (*A. cepa* L.) appears to be the most investigated species, with four studies focusing on the melanogenesis inhibitory property of this species, followed by garlic (*A. sativum* L.), *A. atrovioleceum* Boiss., and *A. paniculatum* L., whose biological effectiveness has been addressed in two different studies. As regards the active constituents identified in investigated *Allium* species, they mainly belong to the sulfur compounds, with six molecules investigated for their inhibitory properties on tyrosinase enzyme and melanogenesis. This prevalence could be related to the abundance of this class of compounds in the *Allium* genus. The remaining compounds mainly belong to flavonoids, one of the classes of natural compounds mainly investigated for their ability to affect melanogenesis.

The studies found in the literature report an interesting biological potential for several species investigated. Overall, however, the mechanisms underlying tyrosinase inhibition and the enzyme kinetics have not been addressed and clarified. Those important aspects need to be more deeply explored.

The in vitro evaluation of the anti-melanogenic properties is commonly performed using mushroom tyrosinase, generally purified from the common edible mushroom *Agaricus bisporus* [108,109]. However, as discussed by Burger and colleagues, the use of mammalian tyrosinase should be more proper than the mushroom one for in vitro assays, as the inhibitors affinity for the mammalian enzyme is usually lower than that for the mushroom tyrosinase. Therefore, active inhibitors of mushroom tyrosinase could not induce the same effects once in contact with the mammalian enzyme. Even if some studies have been performed using crude extracts of human melanocytes as the enzyme source, in many cases, further in-depth research is needed [107].

Clinical effectiveness of topical garlic and onion extracts in dermatological diseases remain to be deeply explored. As reported by Paziar and colleagues, some adverse reactions related to garlic topical use potentially include contact dermatitis and allergic contact dermatitis, but the authors underlined that the topical application of garlic extracts could potentially be effective on a variety of conditions, such as psoriasis, alopecia areata, wound healing, viral and fungal infections, and skin aging [110]. The effectiveness of an onion extract gel on the appearance of postsurgical scars has also been reported [111], and the use of crude onion juice was investigated as an effective topical therapy for patchy alopecia areata [112].

Because of their multi-component nature, plant extracts often exhibit synergistic or additive effects. The combined effects of botanicals or of different molecules from the same plant source may be due to different mechanisms, such as the action on different targets or the enhancement of the solubility or the bioavailability of some constituent consequent to the interactions with each other [113].

Even if the studies included in this review focusing on *Allium* species do not focus on these aspects, many works take into account potential synergistic effects that might occur when combining botanicals. Wang and colleagues, for example, studied the interesting synergistic promotion on tyrosinase inhibition by antioxidant compounds. Ultraviolet radiation, to which human skin is exposed, produces reactive oxygen species (ROS), which in turn activate a variety of biological responses, including melanogenesis. In view of the relationship existing between antioxidant defense systems and melanogenesis, and the synergistic effect which increases the effectiveness of antioxidants in scavenging free radicals while tyrosinase inhibitors reduce melanin production, the authors investigated the potential synergistic effects of glabridin, resveratrol, oxyresveratrol, and phenylethylresorcinol. They demonstrated that the two antioxidant agents resveratrol and oxyresveratrol had a synergistic inhibitory effect on tyrosinase activity [114]. The synergistic effects of many types of natural compounds have been reported. For example, Siridechakorn and coworkers evaluated the synergistic effects of arbutin and kaempferol-7-O-α-l-rhamnopyranoside from *Nephelium lappaceum* L. on melanin inhibition in B16F10 melanoma cells and tyrosinase inhibition [115]. Similarly, Martínez-Gutiérrez and colleagues suggested that the combination of retinol, diosmin, and ferulic acid could be an effective synergistic complex for the treatment of melasma by regulating skin hyperpigmentation [116]. Moreover, Ha and Le, suggested improving the anti-tyrosinase activity by combining different herbal products [117].

Overall, though some negative outcomes have been also detected in the literature, the studies described offer a new perspective on a further potential pharmacological application of *Allium* extracts and phytochemicals, and highlight a new aspect of a well-known and interesting plant genus, which deserves further investigation in the near future.

## Figures and Tables

**Table 1:** Extracts from *Allium* spp. with inhibitory effects on melanogenesis.

Species	Plant Part	Extract	In Vitro Model	Results	Ref.
*A. ampeloprasum* L.	Flowers, leaves, bulbs	MeOH extract	Mushroom tyrosinase	Enzyme inhibitory potential with IC_50_ values = 207.85, 313.40, and 348.10 μg/mL	[58]
*A. ascalonicum* L.	Shallots from 14 cultivation sites in Thailand	Aqueous and EtOH extracts	Mushroom tyrosinase and B16-F10 melanoma cells	At the concentration of 1 mg/mL, the extracts showed about 10–15% tyrosinase inhibition. An optimized shallot extract from fresh shallots decreased melanin synthesis in B16F10 cells in a concentration-dependent manner without affecting cell viability.	[59]
*A. atrovioleceum* Boiss.	Bulb, stem, flower	MeOH extract	Mushroom tyrosinase	The most effective sample, collected in Kemalpaşa, İzmir, Turkey, caused enzyme inhibition, with IC_50_ values equal to 62.53, 67.40, and 78.83 μg/mL for the different extracts.	[60]
	Aerial parts, bulb	MeOH extract, water infusion	Mushroom tyrosinase	TIA, with MeOH extracts (43.86 and 43.44 mg KAE/g for the aerial parts and bulb, respectively) showing higher activity compared to the water extracts	[61]
*A. cappadocicum* Boiss. and Balansa	Aerial parts, bulb	MeOH extract, water infusion	Mushroom tyrosinase	TIA, with MeOH extracts (48.63 and 49.51 mg KAE/g for the aerial parts and bulb, respectively) showing higher activity compared to the water extracts	[61]
*A. cepa* L.	Dried skin	MeOH extract	B16 mouse melanoma cells	Concentration-dependent inhibition of the melanin production	[62]
	n.s.	Onions were autoclaved and fermented with *Saccharomyces cerevisiae* and then extracted with MeOH	Murine melanoma B16F10 cells	The treatment at a concentration of 100 μg/mL for 24 h decreased the protein level of cellular tyrosinase to 65.82%.	[63]
	Outer dry skins and basal and apical trimmings of red onions bulb	80% MeOH extract then partitioned with different solvents	Mushroom tyrosinase	Effective TIA. The 80% aqueous methanol extract was the most effective sample (IC_50_ = 38.9 μg/mL), followed by 80% aqueous ethanol and diethyl ether fractions (IC_50_ = 40.8 and 48.3 μg/mL).	[64]
	Inner layers of white, yellow, and red cultivars and Borettane onions	Juices and distillates	Mushroom tyrosinase	White onion distillate, red onion juice, and yellow onion juice showed the best TIA, with inhibition values equal to 41%, 37%, and 37%, respectively.	[65]
*A. chinense* G.Don	Bulb	Saponin fraction isolated from a 60% EtOH extract	B16 cells	Inhibition of the tyrosinase activity and decreased melanin biosynthesis	[66]
*A. eldivanense* Özhatay	Aerial parts	MeOH extract	Mushroom tyrosinase	Promising inhibitory effect, with IC_50_ = 11.87 µg/mL	[67]
*A. galanthum* Kar. and Kir.	Bulb and chives	Absolute ethanol, 70% ethanol, 50% ethanol, and water extracts obtained by ultrasound-assisted maceration. Diethyl ether extracts	Mushroom tyrosinase and murine tyrosinase (B16F10 cells)	The 50% ethanol and 75% ethanol extracts from the chives significantly inhibited murine tyrosinase as tested on B16F10 cells. The most active murine tyrosinase inhibitor was the diethyl ether extract from the bulb (which induced 82.65% inhibition of mushroom tyrosinase and decreased the activity of murine tyrosinase by 54% at 100 μg/mL).	[68]
*A. goekyigitii* Ekim, H.Duman and Güner	Aerial parts, bulb	MeOH extract, water infusion	Mushroom tyrosinase	TIA, with MeOH extracts (51.17 and 49.70 mg KAE/g for the aerial parts and bulb, respectively) showing higher activity compared to the water extracts	[61]
*A. hirtovaginatum* Kunth	Aerial parts, bulb	MeOH extract, water infusion	Mushroom tyrosinase	TIA, with MeOH extracts (49.53 and 46.50 mg KAE/g for the aerial parts and bulb, respectively) showing higher activity than the extracts obtained through infusion	[61]
*A. ilgazense* Özhatay	Aerial parts	MeOH extract	Mushroom tyrosinase	Inhibitory effect with an IC_50_ value equal to 64 µg/mL	[67]
*A. isauricum* Hub.-Mor. and Wendelbo	Aerial parts, bulb	MeOH extract, water infusion	Mushroom tyrosinase	TIA, with values ranging from 17.25 to 52.84 mg KAE/g	[61]
*A. kastambulense* Bosse	Aerial parts	Methanol/chloroform 4:1 extract	Mushroom tyrosinase	Inhibitory effects with IC_50_ = 59.17 µg/mL	[69]
*A. lycaonicum* Siehe ex Hayek	Aerial parts, bulbs	*n*-Hexane, methanol, and water extracts (maceration and soxhlet)	Mushroom tyrosinase	The extracts showed inhibitory properties, with the methanolic extracts being the most effective samples (values ranging from 132.39 to 139.95 mg KAE/g).	[70]
*A. nigrum* L.	Bulbs, aerial parts	MeOH extract	Mushroom tyrosinase	Good inhibitory potential, with IC_50_ = 22.31 and 51.66 μg/mL	[71]
*A. olympicum* Boiss.	Aerial parts	MeOH extract	Mushroom tyrosinase	Inhibitory effect with an IC_50_ value equal to 321 µg/mL	[67]
*A. pallens* L.	Bulb, stem, flower	MeOH extract	Mushroom tyrosinase	TIA, with IC_50_ values equal to 54.58, 96.65 and 138.43 μg/mL	[72]
*A. paniculatum* L.	Aerial parts, bulb	MeOH extract, water infusion	Mushroom tyrosinase	The MeOH extracts showed TIA, with values equal to 52.87 and 53.17 mg KAE/g for the aerial parts and bulb, respectively. A lower activity was observed for the extracts obtained through infusion (6.35 and 3.02 mg KAE/g)	[61]
*A. paniculatum* L. subsp. *paniculatum* L.	Bulb, stem, flower	MeOH extract	Mushroom tyrosinase	TIA was detected for the flower and bulb samples (IC_50_ = 73.82 and 139.41 μg/mL, respectively). The stem sample was not effective.	[73]
*A. paniculatum* L. subsp. *villosulum* (Hal.) Stearn	Bulb, stem, flower	MeOH extract	Mushroom tyrosinase	All the extracts were effective, with IC_50_ values ranging from 49.16 to 114.25 μg/mL	[73]
*A. peroninianum* Azn.	Aerial parts	MeOH extract	Mushroom tyrosinase	Inhibitory effect with an IC_50_ value equal to 128 µg/mL	[67]
*A. proponticum* Stearn Et N. *Özhatay* subsp. *proponticum* Stearn Et N. Özhatay	Flowers	Essential oil	Mushroom tyrosinase	TIA, with IC_50_ = 38.22 μg/mL	[41]
*A. sativum* L.	n.s.	80% MeOH extract, garlic processed-form (syrup)	Mushroom tyrosinase	Inhibitory activity was reported (from 90.88% inhibition to higher values per 100 g)	[74]
	Bulb	Multi-herbal formulation also containing *Coriandrum sativum* L., *Curcuma longa* L., *Mentha piperita* L., *Piper nigrum* L., *Syzygium* *aromaticum* (L.) Merr. and L.M. Perry, *Syzygium cumini* (L.) Skeels, *Trigonella foenum-graecum* L., and *Murraya koenigii* (L.) Spreng.	Mushroom tyrosinase	Inhibitory potential on tyrosinase enzyme, with IC_50_ = 252.87 μg/mL.	[75]
*A. scabriflorum* Boiss.	Aerial parts, bulb	MeOH extract, water infusion	Mushroom tyrosinase	TIA, with MeOH extracts (44.89 and 43.73 mg KAE/g for the aerial parts and bulb, respectively) showing higher activity compared to the water extracts	[61]
*A. scorodoprasum* L. subsp. *rotundum* (L.) Stearn	Flower, bulb, stem	MeOH extract	Mushroom tyrosinase	The flower extract showed the highest inhibitory potential on tyrosinase enzyme (55.21 mg KAE/g extract).	[76]
*A. sphaerocephalon* L. subsp. *sphaerocephalon* L.	Bulb, stem, flower	MeOH extract	Mushroom tyrosinase	TIA, with IC_50_ = 65.94, 179.42 and 204.71 μg/mL	[77]
*A. sphaerocephalon* L. subsp. *trachypus* (Boiss. Et Spruner) K. Richter	Bulb and stem	MeOH extract	Mushroom tyrosinase	Tyrosinase inhibition (IC_50_ values = 262.50 and 315.88 μg/mL)	[77]
*A. stylosum* O. Schwarz	Dried bulbs, leaves, flowers	MeOH extract	Mushroom tyrosinase	IC_50_ values equal to 49.87, 75.97, and 170.35 μg/mL were obtained for the most effective samples, collected in Bayramli, Izmir, Turkey.	[78]
*A. subhirsutum* L.	Bulbs, aerial parts	MeOH extract	Mushroom tyrosinase	TIA, with IC_50_ = 49.21 and 63.77 μg/mL	[71]
*A. trachycoleum* Wendelbo	Aerial parts, bulb	MeOH extract, water infusion	Mushroom tyrosinase	TIA, with MeOH extracts (51.23 and 48.70 mg KAE/g for the aerial parts and bulb, respectively) showing higher activity compared to the water infusion extracts	[61]
*A. ursinum* L.	Leaves	Water, 70% EtOH, absolute EtOH extracts	Mushroom tyrosinase	The 70% ethanol extract showed the highest activity (IC_50_ = 0.392 mg/mL)	[79]
*A. vineale* L.	Aerial parts, bulb	MeOH extract, water infusion	Mushroom tyrosinase	The MeOH extracts showed the highest inhibitory properties (49.67 and 48.41 mg KAE/g for the aerial parts and bulb, respectively)	[61]

TIA: tyrosinase inhibitory activity; n.s. not specified; Ref.: reference; B16: mouse melanoma cells; KAE: kojic acid equivalent.

**Table 2:** Secondary metabolites from *Allium* spp. with inhibitory effects on melanogenesis.

Compound	Class of Compounds	Investigated *Allium* Species	In Vitro Model	Results	Ref.
Cycloalliin (**1**)	Sulfur compound	*-*	B16 mouse melanoma cells	Reduced α-MSH -induced melanin levels and both protein and mRNA levels of tyrosinase in B16 cells at 3.8 μM	[82]
			Mushroom tyrosinase	Weak inhibition of mushroom tyrosinase	[82]
Diallyl disulfide (**2**)	Sulfur compound	*-*	B16 mouse melanoma cells	At a concentration of 500 μM, inhibition of melanin formation (15.61%) and tyrosinase activity (24.35%)	[83]
Dimethyl disulfide (**3**)	Sulfur compound	*-*	Mushroom tyrosinase	Inhibitory activity, with IC_50_ value equal to 6.5 mM	[83]
			B16 mouse melanoma cells	At a concentration of 500 μM, inhibition of melanin formation (40.57%) and tyrosinase activity (20.77%)	[83]
2,5-dimethylthiophene (**4**)	Sulfur compound	*-*	B16 mouse melanoma cells	At a concentration of 500 μM, inhibition of melanin formation (15.61%) and tyrosinase activity (35.77%)	[83]
Propyl disulfide (**5**)	Sulfur compound	*-*	B16 mouse melanoma cells	At a concentration of 500 μM, inhibition of melanin formation (14.62%) and tyrosinase activity (24.79%)	[83]
1-Propylmercaptan (**6**)	Sulfur compound	*-*	Mushroom tyrosinase	Inhibitory activity, with IC_50_ = 0.5 mM	[83]
			B16 mouse melanoma cells	At a concentration of 500 μM, inhibition of melanin formation (24.15%) and tyrosinase activity (46.89%)	[83]
Quercetin (**7**)	Flavonoid	*A. cepa* L. (dried skin extract)	B16 mouse melanoma cells	Inhibition of the melanin production (IC_50_ = 26.5 μM)	[62]
Quercetin 4′-*O*-β-glucoside (syn. quercetin 4′-*O*-β-D-glucopyranoside; syn. Spiraeoside) (**8**)	Flavonoid	*A. cepa* L. (dried skin extract)	B16 mouse melanoma cells	Inhibition of the melanin production (IC_50_ = 131 μM)	[62]
			Mushroom tyrosinase	Inhibition of mushroom tyrosinase (IC_50_ values = 4.3 μM and 52.7 μM using L-tyrosine and L-DOPA as substrates, respectively)	[84]
Quercetin-3′-*O*-β-D-glucoside (syn. Isoquercitrin) (**9**)	Flavonoid	*A. cepa* L. (dried skin extract)	B16 mouse melanoma cells	Inhibition of the melanin production (IC_50_ = 38.8 μM)	[85]
			Mushroom tyrosinase	Inhibition of mushroom tyrosinase (IC_50_ values equal to 6.5 μM and 48.5 μM using L-tyrosine and L-dihydroxyphenylalanine as substrates, respectively)	[85]
Quercetin-3, 4′-*O*-diglucoside (**10**)	Flavonoid	*A. cepa* L.	Mushroom tyrosinase	Inhibition of tyrosinase enzyme (IC_50_ = 12.6 μM)	[64]
(3β, 22R, 25S)-spirost-5-en-3yl *O*-6-deoxy-α-L-mannopyranosyl-( 1→4)-*O*-6-deoxy-α-L-mannopyranosyl-(1→4)- *O*-[6-deoxy-α-L-mannopyranosyl-(1→2)]-β-D-gluco pyranoside (**11**)	Spirostane-type steroidal saponin	*A. hookeri* Thwaite (root extract)	Mushroom tyrosinase	Inhibitory activity on mushroom tyrosinase with IC_50_ value = 248.7 μM	[86]
(S)-N-*trans*-Feruloyloctopamine (**12**)	Phenolic compound	garlic skin	B16F10 cells	Decreased α-MSH induced cellular melanin content. Real-time PCR and Western blot analyses demonstrated that it down-regulates mRNA and protein expression levels of tyrosinase, leading to a lower melanin content	[87]
Thiacremonone (2,4-dihydroxy-2,5-dimethyl-thiophene- 3-one) (**13**)	alpha-hydroxy ketone	Heated garlic (*A. sativum* L.) juice treated at 130 °C for 2 h	Mushroom tyrosinase	Inhibition of tyrosinase enzyme, with IC_50_ = 101.931 μg/mL	[88]

Ref.: reference; B16: mouse melanoma cells.

**Table 3:** Experiments on *Allium* spp. extracts with negative outcomes.

Species	Plant Part	Extract	In Vitro Model	Results	Ref.
*A. cepa* L.	Flesh	MeOH extract (maceration)	B16 mouse melanoma cells	Any effect was detected on melanin production even at concentrations up to 250 and 500 μg/mL	[62]
*A. ascalonicum* L.	Peel	Hydroalcoholic extract (ethanol 70%)	Mushroom tyrosinase	No activity was observed	[94]
*A. flavum* L.	Stems, flowers	Hydroalcoholic extracts (ethanol 70%)	Mushroom tyrosinase	Any tyrosinase inhibitory activity was detected	[95]
*A. turkestanicum* Regel.	Bulb	Water extracts obtained by ultrasound-assisted maceration	murine tyrosinase (B16F10 cells)	The extract increased the activity of murine tyrosinase	[68]

Ref.: reference; B16: mouse melanoma cells.

## Data Availability

No new data were created or analyzed in this study. Data sharing is not applicable to this article.

## Abbreviations

The following abbreviations are used in this manuscript:

B16	murine melanoma cell line
DPO	diphenol oxidase
KAE	kojic acid equivalent
L-DOPA	L-3,4-dihydroxyphenylalanine
MEHQ	hydroquinone mono methyl ether
MPO	monophenol oxidase
PPOs	plant polyphenol oxidase
PRISMA	Preferred Reporting Items for Systematic Reviews and Meta-Analyses
ROS	reactive oxygen species
TIA	tyrosinase inhibitory activity
Tyr	tyrosinase
Tyrp1	tyrosinase-related protein 1
Tyrp2	tyrosinase-related protein 2
UVB	ultraviolet B
α-MSH	α-melanocyte-stimulating hormone
